# Correction: Takayama, T. Inorganic Particles Contribute to the Compatibility of Polycarbonate/Polystyrene Polymer Blends. *Materials* 2023, *16*, 1536

**DOI:** 10.3390/ma16206771

**Published:** 2023-10-19

**Authors:** Tetsuo Takayama

**Affiliations:** Graduate School of Organic Materials Science, Yamagata University, Yonezawa 992-8510, Japan; t-taka@yz.yamagata-u.ac.jp; Tel.: +81-238-26-3085

There was an error in the original publication [1] in the description of Equation (14).

The original equation was:

(14) $$f_{0}=\frac{1}{1+\frac{3E}{\left(1+\upsilon \right)\sigma_{y}}}$$

The correct equation is:

(14) $$f_{0}=\frac{1}{1+\frac{E}{4\left(1+\upsilon \right)\sigma_{y}}}$$

All *f*_0_ used in the text were obtained using the equation and the correction does not change the claims or arguments of this paper.

The authors state that the scientific conclusions are unaffected. This correction was approved by the Academic Editor. The original publication has also been updated.
